# Kratom safety and toxicology in the public health context: research needs to better inform regulation

**DOI:** 10.3389/fphar.2024.1403140

**Published:** 2024-06-03

**Authors:** Jack E. Henningfield, Oliver Grundmann, Marilyn A. Huestis, Kirsten E. Smith

**Affiliations:** ^1^ Pinney Associates, Inc., Bethesda, MD, United States; ^2^ Department of Psychiatry and Behavioral Sciences, Johns Hopkins University School of Medicine, Bethesda, MD, United States; ^3^ College of Pharmacy, Department of Medicinal Chemistry, University of Florida, Gainesville, FL, United States; ^4^ Institute of Emerging Health Professions, Thomas Jefferson University, Philadelphia, PA, United States; ^5^ Department of Psychiatry and Behavioral Sciences, Johns Hopkins University School of Medicine, Baltimore, MD, United States

**Keywords:** mitragynine, abuse potential, dependence, toxicology, epidemiology, surveillance, therapeutic effects, neuropharmacology

## Abstract

Although kratom use has been part of life for centuries in Southeast Asia, the availability and use of kratom in the United States (US) increased substantially since the early 2000s when there was little information on kratom pharmacology, use patterns, and effects, all critical to guiding regulation and policy. Here we provide a synthesis of research with several hundred English-language papers published in the past 5 years drawing from basic research, epidemiological and surveillance data, and recent clinical research. This review of available literature aims to provide an integrated update regarding our current understanding of kratom’s benefits, risks, pharmacology, and epidemiology, which may inform United States-based kratom regulation. Recent surveillance indicates there are likely several million past-year kratom consumers, though estimates vary widely. Even without precise prevalence data, kratom use is no longer a niche, with millions of United States adults using it for myriad reasons. Despite its botanical origins in the coffee tree family and its polypharmacy, kratom is popularly characterized as an opioid with presumed opioid-system-based risks for addiction or overdose. Neuropharmacology, toxicology, and epidemiology studies show that kratom is more accurately characterized as a substance with diverse and complex pharmacology. Taken together the work reviewed here provides a foundation for future scientific studies, as well as a guide for ongoing efforts to regulate kratom. This work also informs much-needed federal oversight, including by the United States Food and Drug Administration. We conclude with recommendations for kratom regulation and research priorities needed to address current policy and knowledge gaps around this increasingly used botanical product.

## 1 Introduction

Kratom (*Mitragyna speciosa* Korth.) refers to herbal products derived from the leaves of a tropical tree in the Rubiaceae family that grow throughout Southeast Asia and regions of Africa, China, and India (Brown et al., 2017). Domestic cultivation in Florida and Hawaii was also reported. Kratom products marketed in the United States (US) and globally are diverse. These include dried leaf powder that is used in tea-like decoctions, pressed into pellets or encapsulated, as well as more concentrated extracts that range widely in their content of kratom’s major alkaloid, mitragynine (MG), and other minor alkaloids or metabolites, including 7-hydroxymitragynine (7OHMG) (Prozialeck et al., 2020; Todd et al., 2020; Manwill et al., 2022; Sengnon et al., 2023; Grundmann et al., 2024).

In the United States, kratom products are marketed as dietary ingredients and meet criteria under the Food, Drug and Cosmetic Act for regulation by the United States Food and Drug Administration’s (FDA’s) Office of Dietary Supplements but have yet to be accepted for regulation or provided with any regulatory standards for product purity, ingredients, labeling or warnings (FDA, 2024a). At least six kratom products were submitted to FDA by kratom product manufacturers as new dietary ingredient notifications; all were rejected by FDA (Johnson, 2022).^1^ At this writing, we are not aware of any submissions to FDA of new drug applications containing kratom or kratom derivatives. The FDA regulatory position on kratom evolved over the past decade and as of 22 February 2024, it included the following statement on its kratom website: “kratom is not lawfully marketed as a dietary supplement and cannot be lawfully added to conventional foods” (FDA, 2024).

The safety of kratom products from consumer and public health perspectives is a controversial and dynamic topic, as evidenced by differences in United States Federal agencies’ communications on kratom (FDA, 2017b; FDA, 2018; NIDA, 2022; DEA, 2023; FDA, 2024a). Similarly, leading medical institutions including Johns Hopkins University and Mayo Clinic also differ in their characterization of kratom and its place in public health (Johns Hopkins Medicine, 2020; Mayo Clinic, 2022). Recent research on kratom’s alkaloids, *in vivo* and *in vitro* pharmacology, as well as toxicology and epidemiology research, provides new evidence to help address the aforementioned United States regulatory gaps and unreconciled opinions about kratom and public health. Specifically, more than 200 relevant manuscripts published since 2018--many supported by National Institute on Drug Abuse (NIDA) and Southeast Asia organizations, such as the Centre for Drug Research, Universiti Sains Malaysia, Malaysia (Henningfield et al., 2022d)--address some of the controversies and points of confusion, and are included in this review (Henningfield et al., 2022d).^2^

The present review focuses on key areas of debate related to kratom’s safety, abuse potential, use motivations, and risk/benefit ratio at the United States consumer and public health levels. Areas of research to address important gaps in knowledge and to support the development and implementation of federal regulation of kratom products are identified, as are potential regulatory actions based on available evidence.

## 2 Epidemiology

### 2.1 Prevalence

In the United States, anecdotal reports suggest that kratom commerce and use began in the last quarter of the 20th century but was largely limited to Southeast Asia immigrants and returning United States veterans with no published estimates of kratom use prevalence until the 21st century. In 2015, the Botanical Education Alliance (BEA) survey of kratom vendors estimated three to five million kratom consumers in the United States (BEA, 2016). The major United States surveillance systems tracking trends in recreational substance use and treatment-seeking for “addiction” or substance use disorder (SUD) did not include kratom on their annual reports until 2020^3^. The National Survey on Drug Use and Health (NSDUH) prevalence data suggested approximately 1.7–1.8 million past-year kratom consumers from 2019–2021 (Palamar, 2021; SAMHSA, 2022; 2023a; b;c), while the Researched Abuse, Diversion and Addiction-Related Surveillance (RADARS) system estimated 2.03 million, with lifetime prevalence at 3.4 million based on 2018–2019 data (Schimmel and Dart, 2020). Covvey et al. estimated lifetime prevalence of kratom use at 6.1% based on a 2019 survey (Covvey et al., 2020). Other approaches to estimating kratom use prevalence based on kratom import volume and market data projected more than 10 million current users in 2019 (American Kratom Association, 2019). Together, evidence suggests that the NSDUH and RADARS systems likely underestimate kratom use prevalence, indicating that better methods for estimation of novel product use are needed (Henningfield et al., 2022b; Palamar, 2022).

### 2.2 Frequency of use per day

Frequency of kratom use varies considerably and is influenced by reasons for use. Several online surveys suggest that many people use occasionally for relief of acute pain, whereas others use daily in place of or in addition to caffeinated products for mental acuity, for chronic pain, to self-manage mood disorders and opioid and other drug withdrawal symptoms, and to abstain from drugs (Grundmann et al., 2022; Tobacyk et al., 2022). Among people who use regularly, dosing appears to be 1–3 times per day based on proximal needs or goals, such as pain management or increasing energy, focus, alertness, and to enhance productivity (Garcia-Romeu et al., 2020; Smith et al., 2022d; Smith et al., 2024). Using ecological momentary assessment (EMA), Smith et al. (Smith et al., 2022a; Smith et al., 2022b; Smith et al., 2024) obtained13,401 unique kratom use events reported in real-time across 357 participants during a 15-day assessment period, finding that most use occurs more often within the first half of people’s waking hours, with no evidence of weekend binge use or trends of late-night use (Smith et al., 2024).

### 2.3 Reasons for kratom use

The most commonly reported reasons for use in the United States are generally similar to those reported in Southeast Asia field studies and surveys (Singh et al., 2019b), although these studies do not provide nationally representative estimates of the reasons for use. For example, surveys with a targeted focus on people with opioid use histories (e.g., Coe et al., 2019; Garcia-Romeu et al., 2020), or male sexual health issues (Deebel et al., 2023) may overestimate reasons for use related to such issues, compared to surveys based on convenience sampling of current, active kratom consumers, irrespective of use motivation (Grundmann, 2017; Grundmann et al., 2023). Overall, survey and social media self-report are consonant with the approximately 20,000 consumer comments made to Drug Enforcement Administration (DEA) after its proposal to schedule kratom in the Controlled Substances Act (CSA) in 2016, where myriad reasons for use were described, such as sleep improvement, or symptom management for posttraumatic stress disorder (PTSD), fibromyalgia, depression, insomnia, substance use disorders (SUDs), and pain (DEA, 2016; Smith et al., 2021; Smith et al., 2022c; Smith et al., 2024).

A recent EMA study characterizing patterns of kratom use among long-term and regular (using at least 3 days per week) United States kratom consumers found that most participants took kratom to relieve pain, increase energy, productivity, and focus, and for mood improvement (Smith et al., 2024). Among these long-term consumers who used regularly, a motivation of use for some was to avoid kratom-related withdrawal. Taken together, these findings suggest that most use kratom to feel good generally, and not to get “high” (Smith et al., 2024). Such momentary data suggests that many are using kratom for multiple and diverse reasons to address dynamic needs or desires and which may or may not align with broader use motivations. For instance, while some in this EMA study broadly reported using kratom as a long-term substitute for other substances, discrete kratom use events were driven more by quality-of-life needs. This was evidenced by the fact that nearly one-third of the sample had a broad motivation for using kratom as a long-term drug substitute, but few reported a use event to reduce an acute craving for a substance other than kratom which implies that for some, long-term kratom use successfully attenuated drug craving (Smith et al., 2023a; Smith et al., 2024). Surveys investigating reasons for use also suggest that motivations for trying kratom may be evolving within and across consumers with multiple factors driving use, such as increased energy, recreation, aside from self-management of mood or pain (e.g., Smith et al., 2022b).

Indeed, many consumers initiate kratom use for specific health and wellness indications, but continue to use for broader perceived benefits, such as increasing daily productivity (Smith et al., 2022c; Smith et al., 2023a). An evaluation of social media data with independent raters coding 1,274 Reddit posts showed that some believe kratom to be “life-saving” and helpful, whereas others believed that some products may, over time, become “ruinous” (Smith et al., 2021). This is similar to recent findings that among some long-term consumers, those taking more kratom servings with greater frequency and regularity were more likely than others with more varied use patterns to perceive kratom as “life-saving” but also as “habit-forming,” indicating a complex relationship (Smith et al., 2024). However, many consumers, irrespective of use motivations, have reported perceiving kratom as more effective, tolerable, and with less severe side-effects or risks than FDA-approved medicines; kratom consumers also noted that products are accessible and affordable, and are preferred as “natural” and “dietary” approaches over conventional medicines for their lifestyle needs (DEA, 2016; Grundmann, 2017; Smith et al., 2022c; Grundmann et al., 2023; Smith et al., 2024).

### 2.4 Comment on therapeutic and beneficial use

Reasons for kratom use as documented by self-report surveys and field studies of kratom users (e.g., Singh et al., 2015; Grundmann, 2017) are often taken as proxies for therapeutic usefulness (e.g., Grundmann et al., 2018; Prozialeck et al., 2019). However, it is important to note that the FDA generally reserves the terms “therapeutic” “beneficial,” and “medical” use for FDA-approved drugs, not for dietary ingredients or supplements for which only limited health-related (but not “therapeutic”) claims can be made (FDA, 2005). Neither kratom nor any of its naturally occurring alkaloids were submitted for approval nor approved as medicines by the FDA or other major medicine regulatory agencies worldwide. However, evidence of therapeutic potential and beneficial use of kratom are recognized by the US Department of Health and Human Services (DHHS) and World Health Organization (WHO) (Giroir, 2018; United Nations Commission on Narcotic Drug, 2021). Moreover, FDA approval of a substance or product is not the only basis for determining therapeutic or medical use, as most recently demonstrated by the United States DHHS evaluation of marijuana for therapeutic use and rescheduling (U.S. Department of Health and Human Services, 2023a; U.S. Department of Health and Human Services, 2023b).

In 2024, the United States DHHS demonstrated another approach in its determination that marijuana met criteria for “commonly accepted medical use” (CAMU), with the caveat that this was not a determination of efficacy and safety under the Federal Food, Drug, and Cosmetic Act drug approval standard (US DHHS, 2023a; US DHHS, 2023b). By this analysis, marijuana was found to meet criteria for “CAMU in the United States” thus supporting its recommendation that marijuana should be removed from Schedule I of the United States CSA, which is reserved exclusively for substances that are not deemed CAMU (US DHHS, 2023a; US DHHS, 2023b).

The standard required and achieved for marijuana with this approach by DHHS, which involved FDA, was considerable. It included widespread documentation of clinician-recommended use for a variety of medical conditions in 38 states, the District of Columbia and four United States territories, as well as many randomized clinical trials. Presently, kratom would not meet this standard. Nonetheless, it is important to recognize that for centuries in Southeast Asia, kratom was used as a traditional medicine for its beneficial or therapeutic effects (Singh et al., 2015; Singh et al., 2019a). Analogously, but not meeting the level of evidence documented for marijuana by the United States DHHS, therapeutic and beneficial use of kratom were extensively documented in self-report data from United States consumers as discussed in this review (e.g., Swogger and Walsh, 2018; Prozialeck et al., 2019; Smith et al., 2023a; Grundmann et al., 2023). US DHHS review of FDA’s recommendation to place kratom in Schedule I of the CSA included consideration of the fact that many people use kratom to manage pain and opioid use and that banning kratom could lead to “intractable pain, psychological distress, suicide; transition to proven deadly opioids. transition to other potent or harmful drugs.” (Giroir, 2018). Similarly, in its prereview of kratom for potential international drug control and scheduling, the WHO Expert Committee on Drug Dependence determined that scheduling was not warranted, taking into consideration that evidence for kratom abuse liability in animals and humans was “mixed” and “limited,” and that kratom is “used as a part of traditional medicine in some countries” (United Nations Commission on Narcotic Drugs, 2021).

With the caveat that none of the above should be interpreted as supporting the conclusion that kratom should be considered a therapeutic drug by FDA’s drug approval process standard, the findings do support the conclusion that many kratom consumers in the United States and globally report improved health, wellbeing, and quality-of-life from taking kratom (Grundmann et al., 2019; Sharma and McCurdy, 2021). Such human use patterns, and the preponderance of consumer self-report of efficacy, are consistent with nonhuman animal studies investigating kratom alkaloids to attenuate pain, opioid withdrawal, and other conditions (Harun et al., 2021b; Henningfield et al., 2022d; Grundmann et al., 2023). Such convergence of evidence is unsurprising given the known pharmacology of kratom alkaloids (Smith et al., 2023d).

### 2.5 Recreational use

Although self-report data cited above suggest that much kratom use relates to health and wellbeing, what is often referred to as “recreational use” also occurs (Henningfield et al., 2018; Swogger and Walsh, 2018; Smith et al., 2021; Pont-Fernandez et al., 2023; Smith et al., 2024). This is not diagnostically defined and generally refers to people who occasionally use a substance for nonmedical reasons but who do not meet criteria for dependence or use disorder or withdrawal as used by FDA in recommendations for participants for human abuse potential studies (FDA, 2017a). In practice, distinguishing use to self-manage medical problems (e.g., mood disorders, pain, withdrawal), from use to improve productivity, exercise regimens, cognitive performance and focus, pleasurable mood states and sexual pleasure is not always clear in consumer self-reports of reasons for use (e.g., Swogger and Walsh, 2018; Veltri and Grundmann, 2019; Singh et al., 2020; Adzrago et al., 2022; Deebel et al., 2023).

An additional complication is that some use kratom to manage medical problems and in the same time frame or after the medical problem resolves, continue to use for recreation and general wellbeing reasons (Adzrago et al., 2022; Smith et al., 2022c; Deebel et al., 2023; Smith et al., 2024). The many reasons for kratom use are rarely mutually exclusive and are wide-ranging, in keeping with kratom’s complex pharmacology. Importantly, recreational use is not linked to a distinct altered mental state often regarded as a “high” or to development of use disorders, abuse, or addiction to other substances, even though some (not all) who initiate kratom use may have a polysubstance use history (Smith et al., 2024). Contrarily, kratom was clearly linked with the substitution of CSA-listed stimulants, opioids, and alcohol as a harm-reduction agent (Smith and Lawson, 2017). To date there is no evidence that kratom use serves as a gateway to the initiation or use of potent and reinforcing substances, such as stimulants or opioids.

## 3 Kratom use disorder, withdrawal and addiction

### 3.1 Definitions

In this review and the following discussion, we use the terms “use disorder,” “withdrawal,” and “addiction,” similarly as presented in the American Psychiatric Association’ Diagnostic and Statistical Manual of Mental Disorders, fifth Edition (APA, DSM-5, 2013). Note that the World Health Organization’s 11th edition of its International Classification of Diseases (WHO ICD-11, 2019) is in general harmony but not identical to APA’s DSM-5.

The term “substance use disorder” generally replaces what was referred to as dependence in earlier editions of the DSM and ICD. Typically, an SUD assessment includes substance/substance class specifiers (e.g., alcohol, opioids, and stimulants as in alcohol use disorder, opioid use disorder (OUD), and stimulant use disorder, respectively) to form a substance-specific SUD diagnosis. As discussed in DSM-5: “The essential feature of a SUD is a cluster of cognitive, behavioral, and physiological symptoms indicating that the person continues using the substance despite significant substance-related problems” and includes specific criteria and examples (APA, 2013, *p*. 483). SUDs are not all-or-none but range, based on the number of 11 total symptoms, from mild (two to three symptoms), moderate (four to five symptoms), to severe (>6 symptoms). However, not all symptoms are equal, and patients must be evaluated on a case-by-case basis based on which criteria are endorsed. For instance, craving is a pervasive symptom irrespective of substance, but by itself does not necessarily signal serious harm. Conversely, continued use despite a desire to quit or use in physically hazardous situations provides a clearer signal of clinical severity.

“Addiction” is the more general term used to communicate to the public about SUDs and the risk that a substance may produce potent rewarding and reinforcing effects that may contribute to the development of a harmful SUD, as is the case for fentanyl, cocaine and methamphetamine. Thus, although addiction is not a formal diagnostic term in use by either the APA or WHO, as acknowledged in the DSM-5, addiction may sometimes be used interchangeably by some clinicians in reference to patients with severe SUD. Further complicating interpretation of what addiction means to lay persons, the term is widely used to describe a broad range of behaviors that do not necessarily include substances of abuse (regardless of whether there is associated personal or societal harm). For example, “addiction” to caffeinated beverages may be the most prevalent “addiction” among all psychoactive substances self-reported worldwide, but caffeine use is generally considered relatively benign and overall more beneficial than harmful (Goldstein, 2001; Sweeney et al., 2020), supporting the rationale for caffeine use disorder not being listed as a DSM-5 SUD.

Withdrawal refers to the constellation of “substance-specific problematic behavior change, with physiological and cognitive concomitants, that is due to the cessation of or reduction in heavy and prolonged (i.e., chronic) substance use.” (APA, 2013, *p*. 486). Pharmacologically, withdrawal is presumed to reflect neuroadaptation, tolerance, and a state of physiological dependence caused by substance exposure for a sufficiently long time and level of intake that may be specific to the drug (Brunton et al., 2011). Withdrawal symptoms differ widely across drug classes and the DSM-5 lists the symptoms specific to each. Withdrawal is neither necessary nor sufficient for a SUD diagnosis. For example, fetal exposure and chronic use of medicines with a risk of SUD may produce physical dependence, including withdrawal, without evidence of an SUD. Similarly, as noted by the FDA, “The presence of physical dependence or tolerance (as indicated by a withdrawal syndrome) does not determine whether a drug has abuse potential” (FDA, 2017a, *p*. 4). However, withdrawal is included among potential SUD diagnostic symptoms in the DSM-5 and hence *in the presence of an SUD*, the co-occurrence of withdrawal generally implies a more severe SUD (see also FDA, 2017a).

The foregoing is not to imply that withdrawal in the absence of an SUD is not of concern from a health and wellbeing perspective. Withdrawal (and use to avoid withdrawal, i.e., negative reinforcement) can contribute to perpetuation of use in people who do develop SUDs and can be important to manage, particularly in cases where withdrawal can be seriously debilitating such as with alcohol, sedatives, and opioids (Volkow et al., 2016; Szalavitz et al., 2021). In contrast, most people self-manage their caffeine withdrawal, and although withdrawal can occur following discontinuation of stimulants, benzodiazepines, and many antidepressants, the syndromes were not widely recognized by experts or in drug labeling until the 1990s. This was because most patients did not display debilitating symptoms or syndromes requiring medical assistance when they discontinued their medication use that had been at prescribed levels of intake. As implied by the forgoing, self-reported “addiction” with or without withdrawal is not of equal concern across all substances.

### 3.2 Clinical and nonclinical evidence related to kratom use disorders, withdrawal and addiction

Neither DSM-5 nor ICD-11 defined or listed kratom use disorder or withdrawal. However, “kratom use disorder” (KUD), is increasingly used when a kratom consumer meets several of the same criteria as applied to SUDs more broadly. This is evident in NIDA’s conclusion (with the caveat that more research is needed) that “preliminary data suggest that a minority of people report experiencing kratom-related withdrawal symptoms and a smaller minority report experiencing SUD symptoms related to kratom use” and that withdrawal is generally mild to moderate when it does occur (NIDA, 2022).

Research on KUD and withdrawal advanced considerably in the past 5 years and was reviewed in detail elsewhere in an evaluation of kratom’s abuse and withdrawal potential (United Nations Commission on Narcotic Drugs, 2021; Henningfield et al., 2022d). More recently, the findings of a global forum that included at least one representative of most of the research centers that conducted kratom withdrawal and abuse potential studies since 2018 were published (Henningfield et al., 2023). They concurred that KUD and withdrawal profiles are not identical to opioid or other substance disorders in animal or human studies and were overall of lower severity when they did occur (Henningfield et al., 2023; see also Smith et al., 2023b; Smith et al., 2024). As concluded by Henningfield et al. (2023), more research is needed to better inform potential diagnostic listings in the DSM and ICD. Several studies published since that forum also add to the following summary of the state of the science at this writing (see Smith et al., 2023b).

Animal model data suggest low reinforcing and abuse-related effects of kratom’s major alkaloid, MG, which is sometimes the sole kratom-derived ingredient in marketed kratom products. These animal models are relied upon by FDA, NIDA, and WHO for abuse potential evaluations. Specifically, reinforcing effects were weak in two intravenous MG self-administration studies compared to morphine, heroin, and methamphetamine (Yue et al., 2018; Hemby et al., 2019). Specifically, MG pretreatment of animals that had the opportunity to self-administer morphine or heroin intravenously, resulted in reduced opioid self-administration (Yue et al., 2018; Hemby et al., 2019). Another approach to assessing MG reward, conditioned place preference, suggests that MG is not reliable in producing conditioned placement across studies (Yusoff et al., 2018; Wilson et al., 2020).

Drug discrimination studies indicated that MG can weakly or partially generalize to morphine (Hiranita et al., 2020; Obeng et al., 2021). However it most robustly generalized to a nonscheduled alpha-adrenergic drug, lofexidine, which was approved by the FDA for treating opioid withdrawal, and the over-the-counter cold medicine, phenylephrine (Reeve et al., 2020). These findings are consistent with the characterization of MG’s diverse pharmacology that includes effects at several opioid receptors in addition to agonism at adrenergic, serotonergic, D2-dopaminergic and adenosine receptors as discussed in Section 4.3 and other reviews and studies (Kruegel et al., 2016; Kruegel et al., 2019; Henningfield et al., 2022c; Qu et al., 2023; McCurdy et al., 2024).

7-hydroxymitragynine (7OHMG), a MG metabolite not reliably detected in fresh kratom leaves but results from hepatic metabolism is, at high doses, self-administered intravenously by animals, indicating its reinforcing effects (Hemby et al., 2019). More research is needed on other kratom alkaloids and metabolites, such as speciociliatine, to determine if they contribute to the rewarding or reinforcing effects of kratom, or to the self-reported reasons for use and beneficial effects from human kratom use (e.g., Flores-Bocanegra et al., 2020; Henningfield et al., 2022d; Kamble et al., 2022; Huisman et al., 2023).

At high dosages, relative to typical human consumption, discontinuation of chronic MG administration in rodents can produce withdrawal signs that are generally weaker and not identical to those produced by abrupt discontinuation of chronic opioid administration. In animals which were made physically dependent on opioids by daily opioid administration, pretreatment MG can prevent opioid withdrawal when chronic opioid administration was discontinued. Conversely, opioids that are approved by FDA for treating opioid withdrawal can treat MG withdrawal when chronic administration of MG is abruptly discontinued (Harun et al., 2020; Hassan et al., 2020; Wilson et al., 2020; Harun et al., 2021a; Hassan et al., 2021; Johari et al., 2021; See summary of earlier studies and additional discussion in Henningfield et al., 2022d; Henningfield et al., 2023).

### 3.3 Prevalence of KUD, physical dependence and withdrawal

It is important to understand the prevalence of KUD and kratom withdrawal at the population-level. Although US surveys which evaluated KUD suggest that many kratom consumers do not meet criteria for KUD, or if they do it is of mild severity, and do not seek assistance for KUD or withdrawal, some reported “addiction” or withdrawal and seek medical assistance to manage withdrawal, or to aid cessation of kratom use (e.g., Singh et al., 2016; Grundmann, 2017; Singh et al., 2018; Swogger and Walsh, 2018; Coe et al., 2019; Garcia-Romeu et al., 2020; Smith et al., 2021; Smith et al., 2023b; Smith et al., 2024). A recently conducted EMA study of kratom consumers found that greater frequency of use was modestly associated with a greater number of KUD symptoms and taking kratom to avoid kratom-related withdrawal, but that irrespective of frequency, the mean number of KUD symptoms was less than four across the sample (Smith et al., 2024). In that study, 66.7% of participants who were experienced kratom consumers (93.0% had used for >1 year or more) met KUD diagnostic criteria that was predominantly mild (2 or three out of 11 DSM-5 symptoms) in severity, and most reflective of physical dependence (e.g., tolerance, withdrawal), followed by symptoms of craving and using more than intended (Smith et al., 2024). The foregoing findings were recently extended by an anonymous cross-sectional online survey of kratom consumers which found that 25.5% of respondents met KUD criteria; of these, 61.1% were of mild severity (20.0% moderate, 13.9% severe), with the odds of meeting KUD diagnostic criteria 2.83 times higher in those with SUD or psychiatric health histories (Hill et al., 2024). Similar to Smith et al., 2024, the most frequent symptoms were related to tolerance, withdrawal (Hill et al., 2024).

These are useful snapshots of active kratom consumers, but there is no reliable basis for estimating the actual prevalence of kratom use, or KUD and withdrawal in the United States, or the risk for these to develop among consumers. However, some existing evidence provides a basis for designing studies needed to make more precise estimates. Ideally, future surveys would include alternate approaches to represent the population at large. Additionally, it would be important to obtain nationally representative data addressing recency and frequency of use within and across days, and perhaps proxy data estimating KUD and withdrawal. Overall, the animal research is generally consistent with earlier discussed kratom surveys and clinical studies that did not observe kratom withdrawal-related symptoms following various doses of kratom products (Henningfield et al., 2023; Henningfield et al., 2022d) but more study of KUD and withdrawal is clearly needed.

Consumers who report “addiction” and seek help should be offered treatment following addiction treatment strategies in general (Smith et al., 2022a; Swogger et al., 2022). However, treatment with opioids such as buprenorphine and methadone should be judicious and possibly limited to kratom users with histories of opioid use so as not to establish opioid tolerance, physical dependence, and possibly a new use disorder in people who do not have a current or past OUD (Smith et al., 2022c).

### 3.4 Kratom use and abuse-related risks to users and public health

Proxy data indicative of a current or emerging national drug threat is sometimes inferred from estimates of treatment-seeking for use disorder, addiction, or withdrawal. Such data were used by the DEA to support temporary or “emergency” placement (scheduling) in the CSA. Treatment-seeking estimates for kratom were never reported in the SAMHSA Legacy Drug Abuse Warning Network (DAWN) that collected treatment-seeking and other data suggestive of an emerging drug threat from 1992 through 2011, nor the 2019 reestablished DAWN system that includes data from 53 sentinel hospitals (SAMHSA, 2021; 2022). The Treatment Episode Data Set (TEDS), also conducted by SAMHSA since 1992 (SAMHSA, 2023c) collects data including types of substances and demographics on people seeking treatment for SUD. TEDS did not report kratom treatment-seeking as an emerging problem in any of its reports.

The absence of evidence of abuse-related public health problems and treatment-seeking from these federal surveillance systems should not be interpreted as suggesting there are no problems related to KUD or withdrawal and that treatment-seeking does not occur. Published case report data indicate that such events do occur (e.g., Grundmann, 2017; Garcia-Romeu et al., 2020). However, the apparent low rates of KUD or kratom withdrawal treatment seeking in the United States or globally suggest that kratom’s “addiction” related risks are lower than those associated with many controlled substances such as amphetamine, cocaine, and morphine-like opioids (Smith et al., 2023b). Taken together, (e.g., Grundmann, 2017; Garcia-Romeu et al., 2020), these findings and those from a recent EMA study indicate that some users with KUD may experience withdrawal (Smith et al., 2024). However, as summarized by Smith et al. (2024), even among kratom consumers who had indicators of KUD or withdrawal, it was not accompanied by social or functional impairment, hazardous use, or use despite adverse consequences (Smith et al., 2024). The Smith et al. (2024) data are generally consistent with the conclusions of earlier surveys (e.g., Grundmann, 2017; Coe et al., 2019; Garcia-Romeu et al., 2020; Smith et al., 2023b; Henningfield et al., 2023) and the results of two clinical trials that did not observe withdrawal upon discontinuation of kratom administration (Vicknasingam et al., 2020; Huestis, 2022). Still, systematic study of KUD and withdrawal is clearly needed to address the many ambiguities in diagnostic assessment and guide the development of valid clinical assessment instruments (Henningfield et Smith et al., 2022a; 2023b).

## 4 Epidemiology: safety, risks, associated deaths and public health threat

The most commonly reported adverse effects of kratom consumption are indigestion and nausea, which are generally tolerable, self-resolving, and contribute to limiting consumer intake (Swogger et al., 2022; Smith et al., 2023a). Some frequent kratom users report stronger discomfort and adverse effects (e.g., dizziness, constipation and heart palpitations) that also are generally tolerable and self-resolve (Smith et al., 2023a; Feldman et al., 2023). A smaller fraction of kratom consumers, 0.65% of 7,893 respondents in the 2017 survey, sought medical assistance (Grundmann, 2017). The most serious risks of kratom life-threatening sickness and overdose death may be co-ingested drugs, contaminants (e.g., *salmonella*), and adulterants (e.g., opioids including fentanyl and methamphetamine).

The authors are not aware of publications indicating that kratom contributes to violent behavior or serious crime related to its effects on behavior, distribution, or marketing. There are some reports of potential impairment when driving under the influence of kratom; however, whether these were kratom only or involved multiple substances is uncertain (e.g., Knoy et al., 2014; Wright, 2018; Veltri and Grundmann, 2019; Kedzierski and Mata, 2023; Papsun et al., 2023). Papsun et al. (2023) reported detectable blood MG concentrations in 127 impaired driving investigations; however, there is presently no accepted basis for determining what blood mitragynine concentrations produce impairment. Mean and median concentrations in these cases were 133 and 62 ng/mL, respectively, with a range of 8.8–1,000 ng/mL, lower than those of 541 postmortem cases that had 360 and 120 ng/mL mitragynine, respectively, with a range of 5.4–11,000 ng/mL (Wright, 2018; Veltri and Grundmann, 2019).

A recent direct-observation laboratory-based study of 10 adult kratom consumers did not detect impairment following self-administration of participants’ typical kratom product dose or changes in cognitive performance from (pre-dosing) baseline assessments. Two computerized psychomotor tasks and a validated immersive driving simulator capable of detecting alcohol- and cannabis-related driving impairment did not find evidence of impairment or intoxication on all outcomes assessed (Smith et al., 2023c; Zamarripa et al., 2024). However, this exploratory study only examined the acute effects of kratom at self-selected servings among a small sample of long-term kratom consumers, not kratom-naïve individuals. As kratom consumption increases (particularly in public consumption locations such as kratom or kava bars), field sobriety tests coupled with blood plasma levels for MG and other alkaloids are needed in addition to more rigorous PK/PD lab-based assessments.

As discussed below, kratom-associated deaths almost always include the presence of toxic concentrations of other substances. Many reports do not include comprehensive toxicological testing to determine if a kratom-associated death was likely appropriately attributed to kratom (see the following for recommended types of evidence to support determinations of causality in substance related deaths: Gudin et al., 2013; Ossiander, 2014; Davis et al., 2020; Davis and Fligner, 2023). Kratom may be found onsite at an opioid fatality due to its use in managing OUD and withdrawal. Also, intake of novel psychoactive substances (NPS) including designer opioids and benzodiazepines may not be identified with routine toxicology testing, requiring additional testing at reference laboratories at additional costs to resource-restricted public forensic testing laboratories.

Papsun et al. (2023) recently reviewed hundreds of MG cases analyzed at NMS Labs, Inc. And noted MG cases involving blood concentrations up to 1,000 ng/mL in living impaired drivers. The authors highlight the co-positivity of MG with other drugs and also suggest that MG blood concentrations greater than 1,000 ng/mL may be more likely to be associated with severe adverse events (AEs) including death, however, to date there are insufficient data to define a fatal MG blood concentration. Papsun et al. also supported the need for comprehensive toxicology testing prior to assigning cause of death (Gudin et al., 2013; Ossiander, 2014; Davis et al., 2020; Davis and Fligner, 2023).

### 4.1 America’s poison centers’ national poison data system (NPDS)

Kratom exposure and injury reports to the NPDS increased over the past decade along with increases in kratom use and, possibly, related to kratom product adulteration and/or contamination (e.g., Henningfield et al., 2022d; Grundmann et al., 2023). The first reports from the NPDS occurred in 2016 in which fifteen kratom-only deaths were reported. However, it is impossible to confirm how many, if any, of these deaths were primarily caused by kratom or to determine the full extent of toxicology testing performed. For example, an emerging category of potential confounding substances that rapidly proliferated since the early 2000s, NPS, of which many carry serious risks of addiction, morbidity and mortality may be contributing to overdose deaths in which kratom was associated (Huestis et al., 2017; Hall and Miczek, 2019; Higgins et al., 2019; Baumann et al., 2020; Crulli et al., 2022). As discussed by Huestis et al. (2017), the possibility that novel psychoactive substances were consumed and may have contributed to overdose deaths is not typically investigated due to the cost of analysis and the constantly changing predominant novel psychoactive substances available. Novel opioid and benzodiazepine novel psychoactive substances are increasingly abused and also present as adulterants in many types of products including kratom (Di Trana et al., 2022; Collins et al., 2023).

In 2021, the NPDS included 1,524 kratom case mentions. Of those, 948 were single exposure reports, of which 67 were associated with major medical outcomes and four associated deaths (Gummin et al., 2022). For comparison, there were 282,880 pharmaceutical and illegal opioid reports, of which 178,714 were single exposures, resulting in 8,324 major medical outcomes and 720 deaths. More specifically, prescription fentanyl had 4,125 case mentions with 2,151 single exposures resulting in 788 major medical outcomes and 33 deaths, and non-prescription fentanyl had 2,902 case mentions with 1,104 single exposures resulting in 276 major medical outcomes and 136 deaths. Nationwide, the Center for Disease Control and Prevention (CDC) National Center for Health Statistics (NCHS) estimated 107,622 drug overdose deaths in the United States during 2021 of which an estimated 80,816 were attributed to opioids (CDC, NCHS, 2022).

We are not aware of the NCHS ever reporting estimates of kratom-associated deaths as contributing to the national drug overdose estimates (CDC, NCHS, 2022). However, as discussed below, the number of kratom-associated deaths is substantially lower than the main reported contributors to United States overdose deaths. Most kratom-associated deaths include the presence of multiple potentially lethal drug concentrations, making the role of kratom difficult to ascertain (Henningfield et al., 2019).

### 4.2 Kratom-associated deaths cited by other organizations

Estimates of kratom-associated overdose deaths vary widely depending upon the assumptions, the year, and the reporting entity. For example, from its launch until July 2018, the NIDA webpage on kratom stated “Kratom by itself is not associated with fatal overdose, but commercial forms of the drug are sometimes laced with other compounds that have caused deaths.” This webpage was revised in 2018, and the most recent webpage at the time of this writing stating: “A very small number of deaths have been linked to kratom products compared to deaths from other drugs. (Kronstrand et al., 2011; Eggleston et al., 2019; Henningfield et al., 2019; Post et al., 2019; United Nations Commission on Narcotic Drugs, 2021; NIDA, 2022).

In 2018, the FDA reported 44 deaths “involving the use of kratom,” including the 9 Swedish Krypton deaths (FDA, 2018), acknowledging that most of these involved polydrug use. For example, the Swedish deaths followed use of a kratom containing product called Krypton that contained lethal doses of *O*-desmethyltramadol that was determined to be the cause of death by Swedish toxicologists in 2011 (Kronstrand et al., 2011).

In 2019, a Centers for Disease Control and Prevention (CDC) report concluded that 91 of 27,338 overdose deaths in 27 states identified kratom among other substances (Olsen et al., 2019). All but seven included documented polysubstance exposure. Regarding the 7 decedents where MG was the only substance positive on postmortem toxicology, the authors cautioned that other substances may have caused or contributed to death but could not be ruled out because of limited toxicology testing. Comprehensive follow-up testing of samples from reported kratom-only decedents revealed that in all cases with available blood the presence of other potentially lethal substances was revealed (Gershman et al., 2019). Thus, the possible contribution of kratom to these polysubstance deaths is unknown.

In contrast to numerical estimates of annual opioid attributable deaths that exceeded 80,000 in the US in 2021 as discussed in Section 4.1, federal agencies have not provided numerical estimates but rather emphasize the relative rarity of kratom only associated deaths, as illustrated in FDA’s February 2024 update of its “FDA and Kratom” website which states as follows “In rare cases, deaths have been associated with kratom use, as confirmed by a medical examiner or toxicology reports. However, in these cases, kratom was usually used in combination with other drugs, and the contribution of kratom in the deaths is unclear.”

### 4.3 Potential pathophysiological basis for kratom-associated deaths

For most substances of abuse that are attributed with a causal role in the more than 107,000 United States drug overdose deaths in 2021, there is at least a general understanding of the pathophysiological basis for lethality based on animal studies and human forensic toxicology (Brunton et al., 2011; Klaasen, 2018; Rohrig, 2019; Levine and Kerrigan, 2020) For example, rapid and profound respiratory depression is a substantial risk with high doses of morphine-like opioids; alcohol poisoning deaths frequently involve coma and asphyxiation as vomit is inhaled; barbiturate-type sedatives can produce profound sedation and respiratory depression; cocaine and methamphetamine can produce tremors followed by flaccid paralysis, loss of vital support, cardiac arrest, and circulatory failure. Thus, it is often possible for medical examiners to determine the likely substance or substances that contributed to death based on toxicology results and a dose-related profile of effects of the substance(s).

Although some organizations warned of opioid-type kratom overdose risks, the main mechanism of opioid overdose, respiratory depression, does not appear to be a substantial risk from kratom due to differences in the mechanisms of action and signaling pathways of MG and 7OHMG. Evidence suggests that this may reflect their relative weak recruitment of beta-arrestin at opioid receptors (e.g., Kruegel et al., 2016; Kruegel et al., 2019; Henningfield et al., 2022c; Qu et al., 2023; McCurdy et al., 2024). As discussed, MG’s relatively weak respiratory effects as compared to morphine-like opioids may also reflect the “balanced agonist effects of diverse kratom alkaloids and metabolites” (Hill et al., 2022; Qu et al., 2023).

Mitragynine has a complex pharmacology including partial agonism at the µ-opioid receptor, antagonism at the 𝜅-opioid receptor and agonism at adrenergic, serotonergic, D2-dopaminergic and adenosine receptors. Although research on kratom alkaloids and metabolites is expanding, most kratom research has heretofore been focused on MG and 7OHMG. Additional research on MG, speciociliatine, pantetheine and the many other kratom alkaloid’s pharmacology is needed to advance our knowledge of the potential adverse effects of kratom and mechanisms of potentially lethal effects (Henningfield et al., 2022d; Annuar et al., 2024; McCurdy et al., 2024).

Presently, there are no published data describing a physiological pathway by which MG or other kratom alkaloids might produce acute lethality in animals in doses many times that of human intake (Henningfield et al., 2022c). However, such pathways by which MG or another kratom alkaloid could produce lethality in nonhuman animals warrant further research (Annuar et al., 2024). In a direct-observation study conducted at NIDA, respiratory depression was not detected after kratom product self-administration at servings of 1.1–10.9 g of whole-leaf material; all vital signs remained within normal parameters and no AEs occurred (Smith et al., 2023c). Similarly, two other recent clinical studies of MG pharmacokinetics did not observe respiratory depression or serious AEs following controlled administration of 53.2 mg mitragynine in a dried kratom leaf powder (Huestis et al., 2024) or 2 g of kratom powder in an oral solution (Tanna et al., 2022).

### 4.4 Lethal dose

As mentioned above, neither the lethal dose of kratom nor any of its constituents is established in humans. Nonetheless, one study led investigators to the conclusion that a greater than 1,000 ng/mL blood MG concentration was associated with an elevated risk of overdose after review of 6,860 blood positive MG cases analyzed by NMS Labs (Papsun et al., 2023). The authors also discussed the fact that most kratom-associated deaths involve polydrug use complicating determination of causation.

In addition to limitations in toxicology testing in kratom-associated overdose deaths, there are not consistent signs and symptoms for kratom-only overdose-associated deaths, and summaries of medical report findings and autopsies reveal a diverse assortment of associated conditions, frequently consistent with the individual’s medical history, use of NPS, and evidence of SUDs (Domingo et al., 2017; Babin, 2018; Henningfield et al., 2019). For example, kratom-associated death in medical examiner and case reports included cardiovascular, hepatic intoxication and seizure, with most not listing a suspected pathophysiological basis for death (see also Papsun et al., 2023).

Part of the apparent difficulty in determining the lethal dose of oral kratom is related to the difficulty of achieving oral lethal doses in animals. Henningfield et al. (2022c) compared the respiratory effects of up to 400 mg/kg oral MG to up to 150 mg/kg oral oxycodone in rats with the study design published by United States FDA scientists for evaluating opioid respiratory effects. MG doses were systematically increased with the goal of achieving lethality or the highest achievable dose due to MG’s limited aqueous solubility and the maximum allowable volume per gastric delivery. Oxycodone administration produced significant dose-related respiratory depressant effects in blood gases including significantly increased carbon dioxide partial pressure and significantly decreased oxygen saturation and pronounced sedation with one death each at 60 and 150 mg/kg. MG had no significant dose-related respiratory depressant or life-threatening effects. Sedative-like effects, milder than produced by oxycodone, were evident at the highest MG dose. Maximum oxycodone and MG plasma concentrations were dose-related. Consistent with MG’s pharmacology that includes partial μ-opioid receptor agonism with little recruitment of the respiratory depressant activating β-arrestin pathway, MG produced no evidence of respiratory depression at doses many times higher than known to be taken by humans. Another approach to assessing respiratory safety examined MG up to 90 mg/kg in mice as compared to morphine (Hill et al., 2022). Mitragynine produced a small non-life-threatening decrease in respiratory rate at 10 mg/kg with no further effect at the higher doses, consistent with the “ceiling effect” of a partial agonist.

There are also reports of potential hepatic toxicity risks at higher kratom doses (e.g., Gurley et al., 2022); however, the contribution of the well-established hepatic toxins acetaminophen and alcohol that may have been heavily used in persons who use kratom to manage pain and SUDs needs to be disentangled. Similarly, seizures were also reported in some kratom users, and it is possible that the stimulant effects of kratom might reduce seizure threshold; however, these reports are rare. Because caffeine and a broad range of over the counter and prescription medications can also lower seizure thresholds (Hill et al., 2015; van Koert et al., 2018; Epilepsy Foundation, 2023) it is not clear at present whether kratom itself carries such risks. These are examples of potential adverse effects that will benefit from FDA review to determine if the evidence supports mandated kratom product warnings and if so, how the warnings should be worded to meet FDA comprehension standards for product labeling.

None of the foregoing should be taken to imply that kratom or MG is without potential as a primary or contributing cause of death, but rather that the human epidemiology, forensic toxicology, and animal studies are consistent with the profile of products with a broader margin of safety and lower overall risk of overdose as compared to the main contributors to the US drug overdose epidemic (Henningfield et al., 2019; NIDA, 2022). We agree with Papsun et al. (2023) that “Current interpretation of MG in a forensic case is subject to a number of confounding factors, including limited chemical stability, appropriate chemical analysis that ensures separation and identification of pertinent alkaloids, the lack of regulation of commercial kratom products and risks of contamination and adulteration, underlying medical conditions, and frequent detection with other substances.” Suggestion of a lethal dose of kratom or MG in humans should be based on the known toxicology or pathophysiological effects of kratom or its constituents and on animal studies of LD50 with appropriate algorithms.

## 5 Kratom safety across product types in the United States

A perennial topic of discussion is whether United States kratom products carry more risks than those in SEA, because reports of kratom-associated deaths and other adverse effects are more frequent in the United States than in SEA. In fact, kratom-related fatalities were reported in SEA, though the role of co-ingested substances, and the failure to quantify MG, as in United States cases, makes discerning causality practically impossible (Davidson et al., 2021; Weiss and Brent, 2023). An overlapping array of kratom product types, extracts, decoctions of varying concentrations, encapsulated powders, and other product forms are marketed in the United States and SEA; however, fresh leaf made decoctions and leaf chewing are common in Southeast Asia but not the United States. It was reported that some marketed and home brewed products in the United States and elsewhere contain substantially higher concentrations of MG and 7OHMG than achievable by typical tea-making like protocols as well as preparations that included, caffeine, cold medications, opioids and other substances (EMCDDA, 2012; Cinosi et al., 2015; Singh et al., 2016; Prozialeck et al., 2020).

In both the United States and SEA, users report adjusting their preparation and or product selection and pattern and frequency of use to achieve their desired effect and apparently self-regulating intake to minimize undesired effects (e.g., nausea). It is not clear that product type is a determinant of total alkaloid intake at any one time or over the course of a day in multiple times per day consumers (Smith et al., 2023d). Whether product or leaf contamination (e.g., with *salmonella*) and adulteration (e.g., with fentanyl and methamphetamine) is more common in the US as opposed to Southeast Asia is not known.

To date, consumers who use mostly leaf powder products (e.g., in capsules or loose) appear to be self-selecting into research studies (Smith et al., 2022d; Smith et al., 2024). Regular routines based on leaf powder may be due to preference, lower cost as compared to formulated products and extracts, or situation-specific needs where the extracts may be less desired during work or school routines, but more desired for episodic pain or recreation (Smith et al., 2023a; Smith et al., 2024). Research on kratom extract product consumption is needed because extracts vary widely in concentrations and relative ratios of alkaloids and other substances and their pharmacokinetics and effects may differ from dry leaf powder preparations (Todd et al., 2020; Manwill et al., 2022; Sengnon et al., 2023; Grundmann et al., 2024).

Whether the difference in reported kratom-associated adverse effects and deaths in the US compared to Southeast Asia is due to product types or reflects actual real-world differences in rates and types of events is unclear because the United States has an extensive umbrella of reporting systems^4^ that is not matched by any Southeast Asia country in which kratom use is common. Thus, it is possible, if not plausible, that there were kratom-associated deaths in Southeast Asia that would be detected by United States type surveillance systems. It is also apparent that within the US, despite the extensive surveillance, many if not most kratom associated adverse effects and deaths involve other substances.

It is possible that there are product categories (e.g., highly concentrated extracts versus powdered leaf filled capsules, versus low concentration extract and home-brewed decoction) differences in the risk of adverse events and kratom-associated deaths (Grundmann et al., 2023). However, none of the United States surveillance systems reported differences in rates of events or deaths across kratom product types, primarily because the type of product was not mentioned or further scrutinized. This does not mean there are no differences in risk, however, the “signal” that there may be such differences may be below the threshold for reliable detection by these surveillance systems. A national regulatory framework, as could be implemented by FDA, could also ensure that surveillance provided product category specific information and this might provide more sensitive and reliable data to inform regulatory evolution if certain product categories were found to be of higher risk and warrant differential labeling or restrictions on marketing and access.

## 6 Public health and response by regulatory agencies at the state and United States national level

In the United States, kratom was primarily marketed as a dietary supplement without regulatory oversight at the state or federal level. As summarized in a report by the Congressional Research Service, from 2014–2015, six states listed MG and 7OHMG as Schedule I substances, and a seventh state listed synthetic MG and 7OHMG as Schedule I substances. As of this writing four of those states are deliberating replacing their Schedule I bans with regulations to ensure consumer access to kratom that meets regulatory standards with variations on the Kratom Consumer Protection Act (KCPA) first established in Utah in 2018. At the time of this writing, 13 states implemented KCPA versions and five other states provide some regulatory oversight, such as minimum age requirements for purchasing (Heflin, 2023).

In August of 2016, the DEA proposed placing MG and 7OHMG in Schedule I of the CSA by its temporary scheduling authority.^5^ In September 2016, the DEA withdrew its proposal out of concerns about the health risks of a kratom ban expressed by consumers, scientists, and policymakers, and its own reconsideration of the evidence (DEA, 2016; Heflin, 2023).

In November 2017, the FDA proposed permanent Schedule I placement of MG and 7OHMG to the DEA. In an unusual departure from DEA’s general responsiveness to such FDA requests, as of August 2018, the DEA had not acted on FDA’s proposal and the proposal was formally rescinded by the United States DHHS following its review of the scientific evidence and public health implications. In its rescission letter, the Department concluded that kratom did not meet statutory criteria for CSA scheduling and noted that FDA failed to consider the foreseeable public health consequences if kratom was banned and possession criminalized, including risk of “thousands of people” overdosing on opioids if kratom was banned (Giroir, 2018; Heflin, 2023).

NIDA-supported kratom research increased dramatically over the past decade exploring the potential risks and benefits of kratom, including the development of potentially safer new medicines for treating pain, addiction and other disorders with kratom, kratom constituents, and new chemical entities either derived from or similar to kratom constituents (NIDA, 2022). In light of the diverse reasons for kratom use that were summarized in Section 2.3, it is the authors understanding that funding for kratom use may be emerging from other NIH institutes including the National Center for Complementary and Integrative Health, National Institute on Alcohol Abuse and Alcoholism, and National Institute of Mental Health.

DEA continues to monitor kratom, evaluating samples found in drug enforcement operations; however, DEA’s last listing of kratom/MG in its annual National Forensic Laboratory Information System (NFLIS) reports was in 2016 because detection in such cases remained low and relatively stable at about 0.02%–0.03% of all annual substance reports (approximately 1.5–1.9 million) from 2016–2022 (DEA, 2023). DEA never listed kratom or its constituents as a public safety threat in its annual National Drug Threat Assessments, though DEA continues to routinely monitor kratom as a “chemical of concern.”

In part, the absence of crime associated with kratom distribution and marketing is due to the fact that most kratom sold in the United States is marketed by commercial companies and sold in retail stores and over the internet. Approximately 95% of the United States kratom supply is grown, ground, and exported from Indonesia (Pain News Network, 2019). Thailand is beginning to develop its own global kratom export potential since it rescinded its prior Schedule I substance designation for kratom in 2021 (The Nation, 2021), and the University of Florida is researching the development of kratom as a potential domestic agricultural crop (University of Florida, 2023).

## 7 Discussion

Kratom availability and use in the United States increased considerably since its apparently more regional and possibly culture-specific use that occurred during the last quarter of the 20th century. Most use appears motivated by health reasons, wellbeing, and for increasing daily productivity and energy (Smith et al., 2024). Considering the absence of regulatory oversight and absence of standardized labeling (e.g., guidance for use, serving sizes and dosing), rates of serious adverse events and deaths associated with kratom use are low compared to many substances that are used largely for recreational reasons (Henningfield et al., 2019; Prozialeck et al., 2019; Prozialeck et al., 2021; Papsun et al., 2023).

### 7.1 Need for regulation at the state and national level

None of the foregoing is to imply that kratom does not carry risks or should not be monitored or regulated. To the contrary, the DHHS scheduling rescission letter (Giroir, 2018) was explicit that kratom was not without risks and that further research was needed to guide appropriate regulation. Similarly, the WHO ECDD called for continuing surveillance. Many United States states also considered these issues and, as of the writing of this manuscript, 16 states passed KCPA laws that vary across states, with 11 providing multifaceted approaches that include standards to prevent the sale of contaminated and adulterated products, to prevent unsubstantiated claims and sale to minors, to require registration of marketers and products, and to mandate some stipulations for product labeling and warnings (Heflin, 2023).

The emerging interest and passage of laws at the state level highlights the importance of a national regulatory framework by FDA which has considerable expertise in all aspects of dietary supplement regulation from product performance standards to labeling and warnings as are summarized in Table 1.

**TABLE 1:** Research Priorities to guide evolving regulation, labeling and general consumer information.

• Expanded chemistry and pharmacology research to better understand the contributions of kratom’s diverse constituents and metabolites on positive and adverse effects to inform regulatory performance standards for product constituent levels.
• Expanded animal toxicology and pharmacology research to better understand potential harmful consequences related to acute and daily dosing and drug interactions.
• Expanded clinical research to better understand the pharmacokinetics and pharmacodynamics of kratom’s major alkaloids and metabolites, beyond MG and 7OHMG, which have heretofore been the major foci of kratom pharmacology research in general. This should include behavioral research to provide appropriate warnings about potential adverse behavioral effects.
• Expanded clinical research to establish serving size recommendations and maximum daily exposure to kratom and its alkaloids that is considered safe.
• Expanded surveillance and epidemiology research to better understand patterns of use and their association with potential benefits and harms and factors that might increase the risks of use such as drug and other dietary substance intake and preexisting health conditions.

In the absence of FDA-guided labeling, articles and guidance that address safety and potential beneficial use emerged in the medical literature and on the internet from clinician/researchers and health organizations (e.g., WebMD, 2020; NIDA, 2022; NIH NCCIH, 2022; Swogger et al., 2022; University of Florida, 2024).

Cautions as recommended by Swogger et al. (2022), and which include not using concurrently with other substances, using no more than is needed to achieve the intended benefit, and talking to one’s healthcare provider about use, all seem in order and prudent until the FDA develops standardized warnings and labeling for all kratom products marketed in the United States by retail stores and online.

### 7.2 FDA regulatory science needs to guide the implementation of kratom product regulation and evolution over time

Research that is relevant to FDA regulatory science needs is vital to ensure that kratom regulation is evidence-based and evolves to address and minimize risks. The advancement of public health related to kratom hinges on FDA guidance. With respect to kratom itself, research has advanced the understanding of kratom chemistry, pharmacology, epidemiology, benefits, and risks considerably over the past 5–10 years. Although most of this research was not designed and conducted for the purpose of guiding FDA kratom product regulation, much of it is relevant as FDA generally takes a comprehensive approach to product regulation and strives to ensure that its actions are evidence-based and in the interests of public health. FDA product regulation in new areas, including new product types, not only needs a sufficient basis to begin the regulatory process, but requires an ongoing pipeline of regulatory science research to guide the evolution of regulation over time as is evident in FDA’s support (often in collaboration with the United States National Institutes of Health) to guide ongoing regulation of food, drug and tobacco products (e.g., FDA, 2022b; FDA, 2023a). Research priorities to address knowledge gaps as well as to support kratom product regulation and kratom-based new medicines are summarized in Table 2.

**TABLE 2:** Regulatory priorities to protect public health.

• Product performance standards including:
• Maximum allowable concentrations of physiologically active constituents and/or content per serving and possibly per container in the case of manufactured extracts
• Standards for testing for maximum allowable levels of potential contaminants to define and assure purity.
• National labeling requirements and format that includes the ingredient listings consistent with requirements for other dietary supplements, and with the levels by percentage and/or the weight per serving size.
• Prohibition of adulteration by any psychoactive substance or drug product, allowing only accepted flavorings and other substances allowed for food products with labeling consistent with manufactured food products such as coffee, tea, and dietary supplements.
• Potential evidence-based and consumer-tested product warning and precautions, for example, the absence of studies of kratom safety during pregnancy and lactation where FDA needs to consider the evidence and develop labeled warnings.
• Prohibition of unauthorized health claims and examples of what types of descriptive marketing statements are allowable.
• Minimum age of procurement of 21 years.
• Identification of manufacturer or marketer with contact information for consumer comments and AE reporting.

The research reviewed here clarifies that there is presently an adequate epidemiological and basic science evidence base to initiate regulation, as is already occurring in many states, and which could be expertly developed by FDA’s Office of Dietary Supplements. FDA is not only monitoring kratom, but its Office of Dietary Supplements undoubtedly learned much through its review of NDINs and interactions with their sponsors. State-level efforts provide examples of consumer-, manufacturer-, and researcher-informed approaches. FDA already contracted research on the safety of single kratom doses and announced requests for research, such as its 2023 request for proposals, in collaboration with the National Institutes of Health, to study the human abuse potential of botanical kratom, presumably using doses identified during their single ascending dose safety study (FDA, 2024b).

It is important to keep in mind that the time from initiation of Phase 1 clinical drug development trial to submission of a new drug application to FDA is typically 10 years or more, at a cost of a billion dollars or more and that most such efforts do not result in approval of the new drug by FDA (DiMasi et al., 2016; Wouters et al., 2020). New drugs that are approved for therapeutic use by FDA are then for use only by prescription. We note that the FDA has recommended development of new drug applications for potential kratom-derived products, including by its botanical drug development pathway. At the time of this writing, we are aware of at least one such effort that has been made public (e.g., atai Life Sciences, 2022; Sparian Biosciences, 2023).

This process will likely be ongoing, and it is plausible that products will be approved, but for prescription use only within the next decade. Thus, that regulatory pathway will not serve the millions of consumers who presently desire regulatory oversight of the various kratom products they purchase and consume (e.g., DEA, 2016; Grundmann, 2017). Similarly, FDA encouraged development of kratom as pharmaceutical products by FDA’s Botanical Drug Pathway program (FDA, 2016). However, that drug development pathway, only yielded two approved products out of the more than 800 Investigational New Drug Applications received prior to 2018, which speaks to the risks, costs, and uncertainties of this pathway as discussed by FDA officials (Wu et al., 2020). Furthermore, the product of a new drug from the botanical pathway, will also likely be a prescription pharmaceutical product that may or may not appeal to currently available botanical product kratom users.

## 8 Conclusion

In the near term, United States kratom consumers would seem most expeditiously served by an FDA approach to regulate all kratom products to ensure that they meet basic standards for purity, product constituent levels, and labeling and warnings to ensure access to product meeting those standards and prohibit legal sales of products that do not meet the standards. It appears that kratom regulation could be most efficiently developed for the protection of public health by the same type of rulemaking protocols that FDA uses for other dietary products (FDA, 2020). This process includes opportunities for comment and input by diverse stakeholders (including consumers, researchers, manufacturers and public health experts) to ensure that the resulting rule is guided by the latest science, is in the interests of public health, serves consumers, and is practically viable from an industry perspective. Ongoing kratom research, as is already underway with NIH support, helps ensure continuing input into the evolution of consumer and public health evidence-based kratom regulation (NIDA, 2022).

## Footnotes

1 New Dietary Ingredient Notifications (NDINs) can be accepted by FDA or rejected, but not approved. Acceptance is based primarily on the basis of whether the specific product as submitted, including its packaging, level or concentration of ingredients per specified serving size “will be reasonably expected to be safe under the conditions of use recommended or suggested in the labeling” (FDA, 2023b). The NDIN process is distinct from the drug approval process in which new drug applications are subject to review for approval based on therapeutic claims considering the benefit to risk determination supported by clinical studies (FDA, 2022a).

2 NIDA is the lead kratom research supporting agency in the United States. See NIDA’s overview of research and key kratom facts (NIDA, 2022). NIDA’s research funding is also encouraged by legislation from the United States Congress that included language and funding in 2022 and 2023: 116th Congress Report, second Session House of Representatives, Appropriations Bill 2021; 117th Congress Report, first Session, House of Representatives, Appropriations Bill 2022; 117th Congress Report, second Session, House of Representatives Appropriations Bill 2023 all from the Departments of Labor, Health and Human Services, and Education, and Related Agencies.

3 Such surveys discussed in this review include the NIDA supported Monitoring the Future Survey (MTFS); three systems from the Substance Abuse and Mental Health Services (SAMHSA): Drug Abuse Warning Network (DAWN), National Survey on Drug Use and Health (NSDUH), Treatment Episode Data Set (TEDS); and the Denver Health Researched Abuse, Diversion and Addiction-Related Surveillance (RADARS^®^) System.

4 Major United States reporting systems that provide AE and overdose death data: America’s Poison Centers National Poison Data System (APC -NPDS), FDA Adverse Events Reporting System (FAERS), and several systems by the Centers for Disease Control and Prevention (CDC): National Vital Statistics System (NVSS), Web-based Injury Statistics Query and Reporting System (WISQARSW), and the Wide-ranging Online Data for Epidemiologic Research (WONDER).

5 Note that Schedule I is the only option in the United States for substances with “high” abuse and risk potential but which are not approved for therapeutic use by the FDA. Temporary CSA Schedule I placement (i.e., “emergency scheduling”) by the DEA requires determination that use is substantially due to abuse-related effects and poses a known or imminent threat to public health based on the three public health factors of the CSA, i.e., Factors 4, five and 6. The evidence base for these factors typically rest substantially on major national surveillance systems listed in Footnote 4, and documentation of serious risks such as confirmed overdose deaths, and frequently crime and violence. Permanent Scheduling by the DEA requires evaluation of all eight factors of the CSA to support that recommendation to (e.g., by FDA or a citizen petition) or by the DEA. For drug products that are approved by the FDA, if they pose substantial abuse related risks and warrant CSA control, then the level of control and scheduling based on evaluation of the eight factors of the CSA to guide appropriate placement in one of the four schedules reserved for approved drugs, i.e., Schedules II, III, IV or V (see discussion as pertains to kratom in Henningfield et al., 2022; and more generally in FDA, 2017; DEA, 2023; or Henningfield, Coe, Griffiths et al., 2022a).
